# Supplementary material for: A Hemagglutinin Stem Vaccine Designed Rationally by AlphaFold2 Confers Broad Protection against Influenza B Infection
Source: Viruses. 2022 Jun 14;14(6):1305. doi: 10.3390/v14061305 (PMC9229588; doi:10.3390/v14061305)
Supplement: Supplementary file 1 [file viruses-14-01305-s001.zip › viruses-1728017-supplementary.pdf]

Amino Sequence:B/Brisbane/60/2008

DRICTGITSSNSPHVVKTATQGEVNVTGVIPLTTTPTKSHFANLKGTETRGKLCPKCLNCTDLDVALGRPKCTG
KIPSARVSILHEVRPVTSGCFPIMHDRTKIRQLPNLLRGYEHIRLSTHNVINAENAPGGPYKIGTSGSCPNITNG
NGFFATMAWAVPKNDKNKTATNPLTIEVPYICTEGEDQITVWGFHSDNETQMAKLYGDSKPQKFTSSANGVT
THYVSQIGGFPNQTEDGGLPQSGRIVVDYMVQKSGKTGTITYQRGILLPQKVWCASGRSKVIKGSLPLIGEAD
CLHEKYGGLNKSKPYYTGEHAKAIGNCPIWVKTPLKLANGTKYRPPAKLLKERGFFGAIAGFLEGGWEGMIAG
WHGYTSHGAHGVAVAADLKSTQEAINKITKNLNSLSELEVKNLQRLSGAMDELHNEILELDEKVDDLRADTISS
QIELAVLLSNEGIINSEDEHLLALERKLKKMLGPSAVEIGNGCFETKHKCNQTCLDRIAAGTFDAGEFSLPTFDS
LNITAASSGR

Amino Sequence:B60-Stem

MDRICTGITSSNSPHVVKTATQGEVNVTGVIPLTTTPTGSANKSKPYYTGEHTKTTGNSPIWVCTPLKLANGTK
YGSAGSATQEAINKITKNLNSLSELEVKNDQRSSGAMDEDHNEILELDEKVDDLRADTICSQIELAVLLSNEGII
NSEDEQGTGGGYIPEAPRDGQAYVRKDGEWVLLSTFLHHHHHHHHH

Amino Sequence:B60-Stem-8033

MDRICTGITSSNSPHVVKTATQGEVNVTGVIPLTTTPTGSAKIGTSGSCPNITNGNGFFATMAWAVPKNGSAQ
MAKLGSALPQSNKSKPYYTGEHTKTTGNSPIWVCTPLKLANGTKYGSAGSATQEAINKITKNLNSLSELEVKN
DQRSSGAMDEDHNEILELDEKVDDLRADTICSQIELAVLLSNEGIINSEDEQGTGGGYIPEAPRDGQAYVRKDG
EWVLLSTFLHHHHHHHHH

Amino Sequence:B60-Stem-12G6

MDRICTGITSSNSPHVVKTATQGEVNVTGVIPLTTTPTGSAPYKIGTSGSCPNITNGNGFFATMAWAVPKNDGG
GGSHSDDETQMAKLGGGGSLPQSGGSANKSKPYYTGEHTKTTGNSPIWVCTPLKLANGTKYGSAGSATQEA
INKITKNLNSLSELEVKNDQRSSGAMDEDHNEILELDEKVDDLRADTICSQIELAVLLSNEGIINSEDEQGTGGGY
IPEAPRDGQAYVRKDGEWVLLSTFLHHHHHHHHH

Amino Sequence:B60-Stem-SD84

MDRICTGITSSNSPHVVKTATQGEVNVTGVIPLTTTPTGSARLSTHNVINAENAPGGPYKIGTSGGGGSNGVTT
GSANKSKPYYTGEHTKTTGNSPIWVCTPLKLANGTKYGSAGSATQEAINKITKNLNSLSELEVKNDQRSSGAM
DEDHNEILELDEKVDDLRADTICSQIELAVLLSNEGIINSEDEQGTGGGYIPEAPRDGQAYVRKDGEWVLLSTFL
HHHHHHHHH

Amino Sequence:B60-Stem-46B8

MDRICTGITSSNSPHVVKTATQGEVNVTGVIPLTTTPTGSAAKSLNGGGGSGSLPLIGGSANKSKPYYTGEHTK
TTGNSPIWVCTPLKLANGTKYGSAGSATQEAINKITKNLNSLSELEVKNDQRSSGAMDEDHNEILELDEKVDDL
RADTICSQIELAVLLSNEGIINSEDEQGTGGGYIPEAPRDGQAYVRKDGEWVLLSTFLHHHHHHHHH

Amino Sequence:B60-Stem-8071

MDRICTGITSSNSPHVVKTATQGEVNVTGVIPLTTTPTKSHFANLKGTETRGKLCPKCLNCTDLDVALGRPKCT
GKIPSARVSILHEVRPVGSAGSLPLIGEADCLHEKYGGLNKSKPYYTGEHTKTTGNSPIWVCTPLKLANGTKYG
SAGSATQEAINKITKNLNSLSELEVKNDQRSSGAMDEDHNEILELDEKVDDLRADTICSQIELAVLLSNEGIINSE
DEQGTGGGYIPEAPRDGQAYVRKDGEWVLLSTFLHHHHHHHHH

Figure S1. Construct design and diagrams of HA stem. Sequences of full length, B60-Stem, B60-Stem-8033(blue), B60-Stem-12G6(yellow), B60-Stem-SD84(purple), B60-Stem-46B8(red) and B60-Stem-8071(yellow).

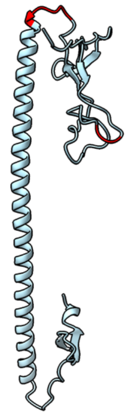

Figure S2. The prediction model of B60-Stem monomer by AlphaFold2. GSA linker was colored red.

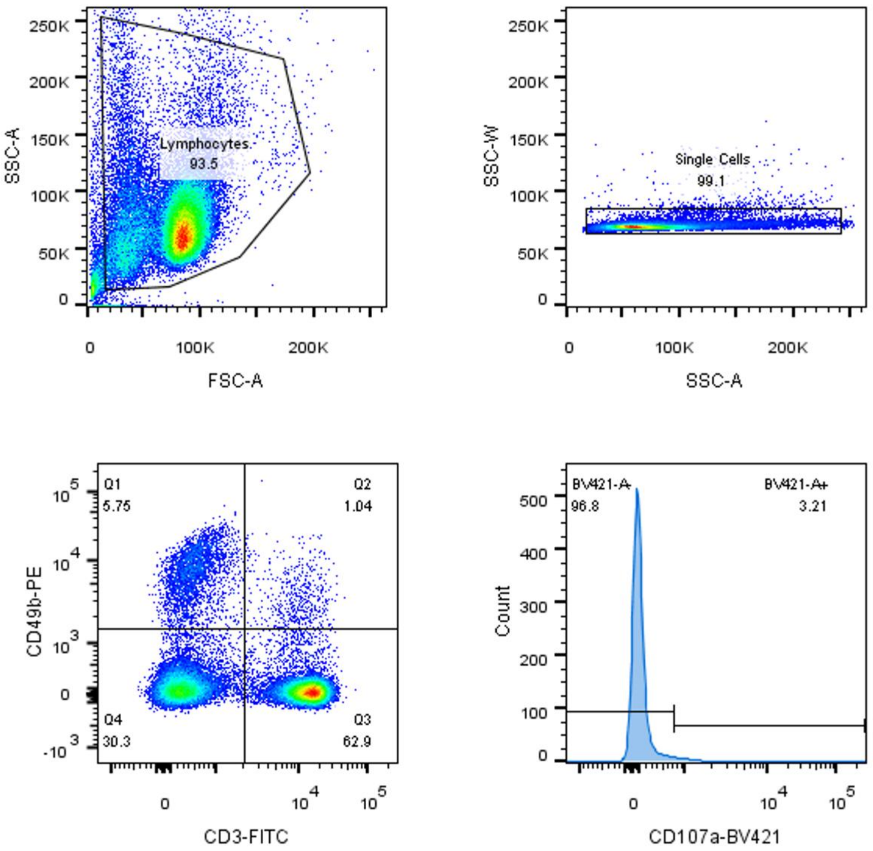

Figure S3. The gating strategy of NK cell activation by FACS. (A) PBMCs isolated from NC group mice, and the cells were stained with anti-mouse antibody FITC-antiCD3, PE-antiCD49b and BV421-antiCD107a.

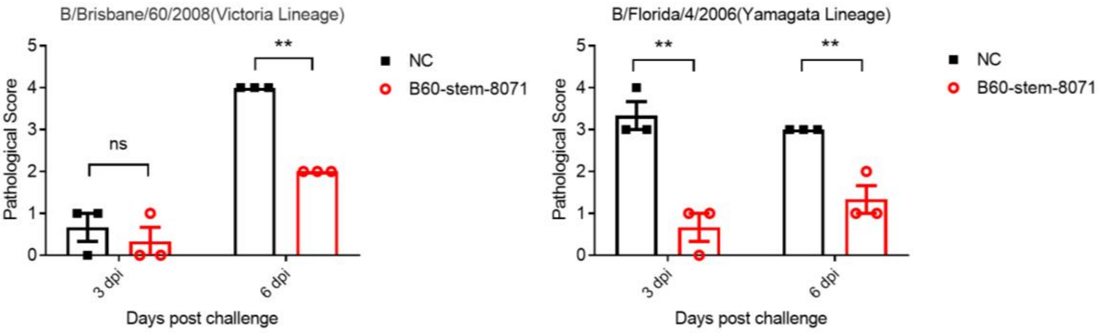

Figure S4. Histological scoring is shown as mean ± SEM (n = 3 mice) with asterisks representing significant differences Significance (*p < 0.05, **p < 0.01).

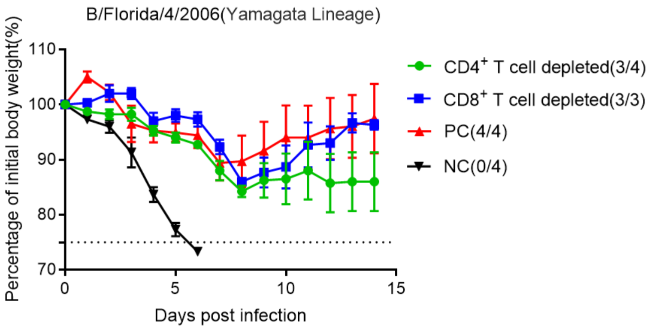

Figure S5. Body weight loss of mice vaccinated with B60-stem-8071 were treated with anti-CD4$^+$ (in green) or anti-CD8$^+$ T cells antibodies (in blue) after intranasal infection with B/Florida/4/2006.
